# Large-scale structure-informed multiple sequence alignment of proteins with SIMSApiper

**DOI:** 10.1093/bioinformatics/btae276

**Published:** 2024-04-22

**Authors:** Charlotte Crauwels, Sophie-Luise Heidig, Adrián Díaz, Wim F Vranken

**Affiliations:** Interuniversity Institute of Bioinformatics in Brussels, ULB-VUB, Brussels, 1050, Belgium; Structural Biology Brussels, Vrije Universiteit Brussel, Brussels, 1050, Belgium; AI Lab, Vrije Universiteit Brussel, Brussels, 1050, Belgium; Interuniversity Institute of Bioinformatics in Brussels, ULB-VUB, Brussels, 1050, Belgium; AI Lab, Vrije Universiteit Brussel, Brussels, 1050, Belgium; Evolutionary Biology & Ecology, Université libre de Bruxelles, Brussels, 1050, Belgium; Interuniversity Institute of Bioinformatics in Brussels, ULB-VUB, Brussels, 1050, Belgium; Structural Biology Brussels, Vrije Universiteit Brussel, Brussels, 1050, Belgium; AI Lab, Vrije Universiteit Brussel, Brussels, 1050, Belgium; Interuniversity Institute of Bioinformatics in Brussels, ULB-VUB, Brussels, 1050, Belgium; Structural Biology Brussels, Vrije Universiteit Brussel, Brussels, 1050, Belgium; AI Lab, Vrije Universiteit Brussel, Brussels, 1050, Belgium

## Abstract

**Summary:**

SIMSApiper is a Nextflow pipeline that creates reliable, structure-informed MSAs of thousands of protein sequences faster than standard structure-based alignment methods. Structural information can be provided by the user or collected by the pipeline from online resources. Parallelization with sequence identity-based subsets can be activated to significantly speed up the alignment process. Finally, the number of gaps in the final alignment can be reduced by leveraging the position of conserved secondary structure elements.

**Availability and implementation:**

The pipeline is implemented using Nextflow, Python3, and Bash. It is publicly available on github.com/Bio2Byte/simsapiper.

## 1 Introduction

Proteins execute a myriad of functions based on combinations of 20 different types of amino acids arranged in distinct sequences, which can adopt a variety of 3D shapes. The investigation of protein function, and studies of how they evolved, rely largely on the analysis of Multiple Sequence Alignments (MSA) of proteins with a common evolutionary origin (O’Sullivan *et al.* 2004, Carpentier and Chomilier 2019). The evolutionary selection pressure on a protein is indeed driven by its ability to function correctly, with even drastic changes in a protein’s amino acid sequence tolerated as long as its overall behavior and shape maintains its function. Sequence-based alignment methods, while being very fast, struggle with the alignment of protein families with high structural and functional conservation but low sequence identity (SI) (<30%) (O’Sullivan *et al.* 2004, Carpentier and Chomilier 2019, Rajapaksa *et al.* 2023), such as RNA Recognition Motifs (RRMs) (Roca-Martínez *et al.* 2023), membrane proteins like G-Protein Coupled Receptors (Zhou *et al.* 2019) or designed proteins with no evolutionary connection (Figueroa *et al.* 2013, Huang *et al.* 2016). Moreover, sequence-based algorithms are optimized for single globular protein domains and lose accuracy when confronted with larger proteins with multiple domains (Santus *et al.* 2023). For these reasons, structure-informed MSA methods have provided the “gold standard” MSAs that serve to evaluate sequence-based alignment algorithms (Carpentier and Chomilier 2019).

The main challenge for structure-informed MSA methods has been a lack of available protein structures (Chatzou *et al.* 2016), which is now resolved through the emergence of accurate AI-based prediction of protein structure from sequence (Jumper *et al.* 2021, Lin *et al.* 2023): over 200 million protein structure models are now available through the AlphaFold Protein Structure Database (AF2 DB) (Varadi *et al.* 2022). Methods such as ColabFold (Mirdita *et al.* 2022) and ESMFold (ESMF) (Lin *et al.* 2023) can also provide protein structure models of reasonable accuracy within minutes. Even when such predicted protein structures are of reduced accuracy, they can significantly improve structure-informed MSAs (Baltzis *et al.* 2022, Rajapaksa *et al.* 2023).

For the structural alignment of larger datasets, several challenges are present. Access to structural information and/or the prediction of models can be time-intensive. Moreover, the computational resources required to find the optimal alignment grow exponentially with the number of proteins to align (Rubio-Largo *et al.* 2018, Lladós *et al.* 2021, Santus *et al.* 2023). For the efficient study of protein families, this process of structure-informed MSAs requires streamlining.

We introduce SIMSApiper, an automated pipeline to create structure-informed MSAs of protein sequences not limited by the size of the dataset nor by the SI between the protein sequences. SIMSApiper combines existing tricks and tools of current MSA workflows into a single framework implemented in Nextflow (Di Tommaso *et al.* 2017) to maintain reproducibility, scalability and convenience across users and platforms. The protein sequences to be aligned are divided into subsets, either provided by the user or automatically created based on SI cutoffs. This significantly reduces compute times and allows the method to scale to thousands of protein sequences. The required protein structures can be provided by the user and complemented with models from the AF2 DB or ESMF. Each subset is aligned using T-Coffee’s tool 3DCoffee (O’Sullivan *et al.* 2004) (referenced to as T-Coffee hereafter) and the TMalign and SAP algorithms (Taylor 2000, Zhang and Skolnick 2005). These sub-MSAs are then merged into the final MSA using MAFFT (Katoh *et al.* 2019), with a final secondary-structure based refinement step reducing the number of gaps. SIMSApiper’s accuracy was tested on three datasets, a subset of HOMSTRAD, and the TIM-barrel and GroEL protein families. The quality of SIMSApiper’s and T-Coffee’s alignments are comparable on challenging datasets. However, SIMSApiper is faster than T-Coffee on larger datasets (e.g. 20 times faster on 350 TIM-barrels proteins).

## 2 Materials and methods

### 2.1 Computation

SIMSApiper is implemented in Nextflow, with dependencies provided in containers compatible with Docker (Merkel *et al.* 2014) and Singularity(Kurtzer *et al.* 2017), ensuring reproducibility (Conte *et al.* 2023) and ease of use. Nextflow enables parallelization, produces extensive log files and provides check points from which execution can be resumed, all properties that contribute to the decrease in compute time while optimizing the use of compute resources.

**Figure 1.**
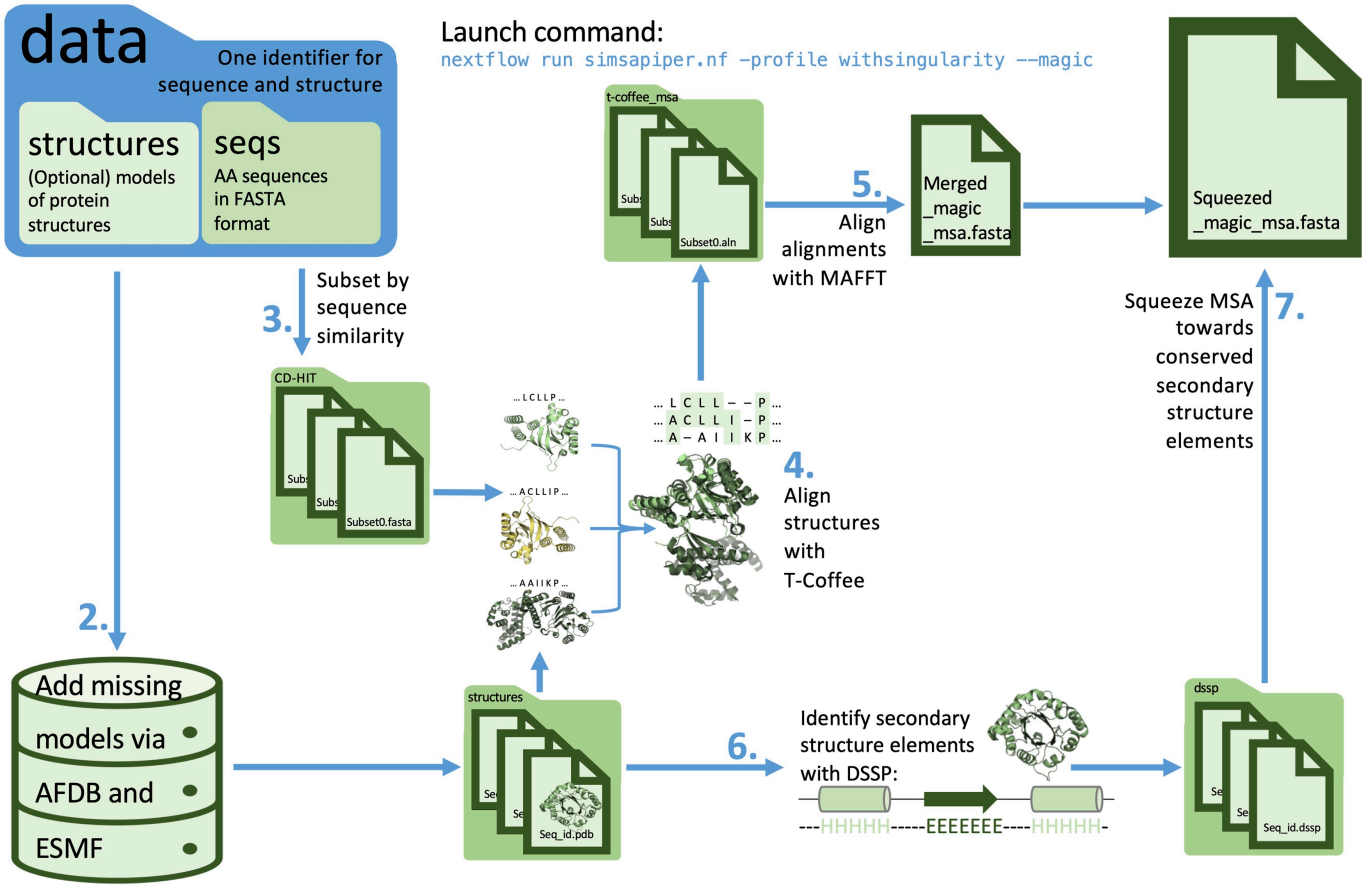
Simplified workflow of data handling and integrated tools in SIMSApiper pipeline.

SIMSApiper requires only a sequence file to run. The input sequences can first be cleaned (step 1, shown in Supplementary Fig. S1) to reduce the dataset’s redundancy using CD-Hit (Li and Godzik 2006). We recommend a cutoff of at least 90% SI, with 70% SI likely better to reduce sampling bias for a given protein family. In Fig. 1 step 2, SIMSApiper matches user-provided structures to the sequences and automatically collect structure models for the remaining ones. For sequences labeled with their corresponding Uniprot ID (UniProt Consortium 2023), models are automatically collected from the AF2 DB. Sequences shorter than 400 residues are submitted to the ESMF online resources to generate a model. Finally, sequences not matched to a structure after this step are called *structureless*. In step 3, similar sequences, with a matched structure, are grouped together into subsets. These can be based on user-provided sequence files or generated automatically using CD-Hit with a SI cutoff as low as 20%. Sequences that are too different can be collated with small clusters, thereby creating a minimal total amount of subsets. Alternatively, they can be labeled as *orphan*. These and the *structureless* sequences are not aligned in the next step and will be integrated later. Each sequence subset along with the corresponding structures are submitted to T-Coffee (step 4) for alignment with TMalign and SAP. Subsets can be aligned in parallel if the required hardware is available. After aligning each subset, the subset MSAs are submitted to MAFFT (step 5) with the “merge” setting, which preserves each subset MSA’s shape and therefore retains the structure information. The *orphan* and *structureless* sequences are then individually aligned to the main alignment to generate one final alignment. As step 5 tends to dramatically increase the number of gaps in variable and hard to align regions such as loops, SIMSApiper provides the option to reduce gaps by squeezing the alignment towards DSSP defined conserved secondary structure categories (steps 6,7) such as β-strands or α-helices (Kabsch and Sander 1983). This step is based on the principle that the (spatial) position of amino acids relative to conserved secondary structure elements is the most relevant, not their SI (Roca-Martínez *et al.* 2023).

### 2.2 Performance evaluation

The overall quality of the MSA is quantified using the Column Score (CS). For each column of the query MSA (i), a score of 1 is assigned when all residues are aligned as in a reference MSA, a score of 0 is given if any misalignment is present. The number of matching columns between (regions of) the MSAs is then divided by the total number of evaluated columns (m) to produce a final score CS (Equation 1).

(1) $$CS=\frac{\sum_{i=1}^{m}score_{i}}{m}$$

### 2.3 Validation datasets

SIMSApiper was evaluated on three datasets:

1. The TIM-barrel protein family [InterPro accession: IPR000652 (Paysan-Lafosse *et al.* 2023)]: 663 sequences with SI ⩾20% and highly conserved structure of 4 βαβα anti-symmetrical units (Wierenga 2001).
2. Similarly to T-Coffee’s validation strategy (O’Sullivan *et al.* 2004), we selected the most demanding MSAs (SI <25% and a size >4 proteins) from the hand-curated HOMSTRAD reference database (Mizuguchi *et al.* 1998), (release date: 07/05/2022). This resulted in 51 MSAs (HOM51) described in detail in Supplementary Fig. S4.
3. The GroEL protein family: Conserved obligate chaperonin across archaea and prokaryotes with a structural homolog in eukaryotes (Ansari and Mande 2018). 50k sequences were collected from Uniprot KB with estimated SI ⩽30%.

### 2.4 Hardware

A detailed description of the used hardware to compute the SIMSApiper validation MSAs is available in Supplementary Table S1.

## 3 Results

### 3.1 Fast and accurate alignment of the TIM-barrel protein family

SIMSApiper automatically cleaned the InterPro TIM-barrels protein family using a >90% SI cutoff, resulting in a dataset of 379 sequences to align. It then grouped the sequences into 6 subsets using a SI cutoff of 30%. Each subset was aligned by SIMSApiper with T-Coffee and required 10 CPUs, 15 GB memory and ran between 1 and 25 min depending on its size (Supplementary Fig. S2). Nextflow enabled efficient resource use in this step by running jobs in parallel, resulting in a total runtime of 33 min. Concurrently, this would result in an approximate runtime of 2h12. If no subsets are created, equivalent to running directly T-Coffee on the 379 sequences, the total runtime increases to 9h48 and requires 45 GB memory instead (Supplementary Fig. S2). This significant increase in computational resources highlights how SIMSApiper can more efficiently accommodate larger datasets than T-Coffee alone, by clustering the input data. From the TIM-barrel SIMSApiper generated MSA, we extracted the 10 sequences that are also present in a manually curated, gold standard, structure-informed TIM-barrel MSA (Maes *et al.* 1999) (MSA_SIMSApiper_ and MSA_ref_, respectively).

A comparison of MSA_SIMSApiper_ and MSA_ref_ using the CS metric (Supplementary Table S2), illustrates that SIMSApiper effectively aligns conserved structured regions (CS = 99,3%), with a lower score for the diverse and unstructured loops (CS = 91.3%). The overall CS (CS = 95.8%) shows SIMSApiper’s capability to generate a quality MSA despite the use of clustering to accommodate larger datasets (step 3). The CS also highlights the importance of the squeezing step (step 6,7), which helps to reduce the number of gaps in the MSA and improving its quality (CS from 85.2% to 91.3% in the loops). Running SIMSApiper without the squeezing mode is equivalent to running T-Coffee. By squeezing, SIMSApiper thus performs slightly better than T-Coffee for this dataset. SIMSApiper was further evaluated by comparing the alignment of two designed TIM-barrels, sTIM 11 (Huang *et al.* 2016), and Octarellin VI (Figueroa *et al.* 2013), to the 10 natural TIM-barrels mentioned above. Despite the low SI (<20%) and different 3D structure (>4Å) between the designed and natural proteins (Supplementary Fig. S3a–d), SIMSApiper identified similar secondary structure elements (β-sheets and α-helices) and effectively aligned those with each other (Supplementary Fig. S3e).

### 3.2 Alignment of the challenging datasets from HOMSTRAD

When comparing the HOM51 MSAs with the equivalent SIMSApiper MSAs, a median CS of 80% is obtained for the conserved structured regions (Supplementary Fig. S5). SIMSApiper can thus successfully align datasets with low SI. As a similar overall median CS is obtained with or without squeezing (53% and 55%, respectively), the squeezing (step 7) does not impact the overall alignment quality in these examples. This is because of the way the HOM51 MSAs are constructed, with many gaps in the loop regions. Squeezing the loops of the obtained SIMSApiper MSAs towards their conserved regions is therefore in this case counter-effective. Nevertheless, because running SIMSApiper with the no squeezing setting is equivalent to running native T-Coffee (no pre-processing), we can conclude that T-Coffee also struggles with aligning these loop regions. In our opinion, because of the sequence and structural diversity of the loops, the alignment of the loops in the HOM51 MSAs is not more accurate than the alignment obtained with T-Coffee, and thus by extension SIMSApiper. Analyzing the CS of the unconserved regions is therefore of limited relevance here.

### 3.3 Alignment of large datasets: the GroEL protein family

We applied a 70% sequence similarity cutoff on the large GroEL dataset to reduce compute times, leaving 1600 diverse proteins to be aligned. Four sequences were excluded due to a high number of unresolved amino acids. For 400 sequences, no matching models could be found in the AF2 DB. As the average sequence length exceeds 400 residues for GroEL, we supplied the structural information with ESMF predictions run on the VSC Tier-2 general-purpose clusters provided by VUB-HPC. Assembling the complete structural dataset took 7 min to retrieve models from AF2 DB and 2.5 h to predict the remaining models locally. Thirty-eight subsets were generated and aligned in parallel. The alignment process took 45 min, including post-processing. Supplementary Figure S6a highlights the final MSAs well aligned secondary structure elements and Supplementary Figure S6b shows that SIMSApiper has maintained the conservation of experimentally confirmed residues for ATP binding (Xu *et al.* 1997, Koike-Takeshita *et al.* 2014), which are crucial for GroEL to perform its function.

## 4 Discussion

The rise of AI-driven protein fold predictors has greatly impacted the field of structural bioinformatics by providing accurate structure models for well-folded protein regions, with their accessibility ensured through both sequence- and structure-based methods [BLASTP (Camacho *et al.* 2009), Foldseek (Barrio-Hernandez *et al.* 2023)] on AF2 DB.

Recent reviews (Rajapaksa *et al.* 2023, Santus *et al.* 2023) highlight the need for a MSA pipeline that can leverage this new data for the study of protein families with low SI but conserved secondary structure. Such approaches will allow to pinpoint similarities and differences, enabling a deeper understanding of their function. SIMSApiper provides this functionality by combining user-provided structures and automatically retrieved models from AF2 DB or ESMF and by enabling the use of the alignment tool T-Coffee, known for generating high-quality MSAs, on larger datasets. The exponential scaling of the resources related to this alignment step is successfully mitigated by SIMSApiper through creating sequence subsets. SIMSApiper generates high quality structure-informed MSAs, which is illustrated by the excellent match with manually curated MSAs and its ability to align designed proteins with low sequence and 3D structural identity but conserved secondary structure elements. The validation further highlights the importance of the pre-processing step subdividing the sequences, as well as the post-processing squeezing step to reduce the computing time and the number of gaps, respectively. SIMSApiper represents not only a convenient way to create MSAs based on experimental and modeled structures, but also, it can significantly reduce calculation times while maintaining the accuracy of the generated structure-informed MSA.

## Supplementary Material

btae276_Supplementary_Data

## Data Availability

The complete dataset and scripts to reproduce the results are available on GitHub (github.com/Bio2Byte/simsapiper).

## References

[btae276-B1] Ansari MY , Mande SC. A glimpse into the structure and function of atypical type I chaperonins. Front Mol Biosci 2018;5:31. 29696145 10.3389/fmolb.2018.00031 PMC5904260

[btae276-B2] Baltzis A , Mansouri L, Jin S et al Highly significant improvement of protein sequence alignments with AlphaFold2. Bioinformatics 2022;38:5007–11. 36130276 10.1093/bioinformatics/btac625 PMC9665868

[btae276-B3] Barrio-Hernandez I , Yeo J, Jänes J et al Clustering predicted structures at the scale of the known protein universe. Nature 2023;622:637–45. 37704730 10.1038/s41586-023-06510-w PMC10584675

[btae276-B4] Camacho C , Coulouris G, Avagyan V et al BLAST+: architecture and applications. BMC Bioinformatics 2009;10:421–9. 20003500 10.1186/1471-2105-10-421 PMC2803857

[btae276-B5] Carpentier M , Chomilier J. Protein multiple alignments: sequence-based versus structure-based programs. Bioinformatics 2019;35:3970–80. 30942864 10.1093/bioinformatics/btz236

[btae276-B6] Chatzou M , Magis C, Chang J-M et al Multiple sequence alignment modeling: methods and applications. Brief Bioinform 2016;17:1009–23. 26615024 10.1093/bib/bbv099

[btae276-B7] Conte AD , Mehdiabadi M, Bouhraoua A et al Critical assessment of protein intrinsic disorder prediction (CAID)-results of round 2. Proteins Struct Funct Bioinf 2023;91:1925–34. 10.1002/prot.26582 37621223

[btae276-B8] Di Tommaso P , Chatzou M, Floden EW et al Nextflow enables reproducible computational workflows. Nat Biotechnol 2017;35:316–9. 28398311 10.1038/nbt.3820

[btae276-B9] Figueroa M , Oliveira N, Lejeune A et al Octarellin VI: using Rosetta to design a putative artificial (β/α)8 protein. PLoS One 2013;8:e71858. 23977165 10.1371/journal.pone.0071858 PMC3747059

[btae276-B10] Huang P-S , Feldmeier K, Parmeggiani F et al De novo design of a four-fold symmetric TIM-barrel protein with atomic-level accuracy. Nat Chem Biol 2016;12:29–34. 26595462 10.1038/nchembio.1966 PMC4684731

[btae276-B11] Jumper J , Evans R, Pritzel A et al Highly accurate protein structure prediction with AlphaFold. Nature 2021;596:583–9. 34265844 10.1038/s41586-021-03819-2 PMC8371605

[btae276-B12] Kabsch W , Sander C. Dictionary of protein secondary structure: pattern recognition of hydrogen-bonded and geometrical features. Biopolym Original Res Biomol 1983;22:2577–637. 10.1002/bip.360221211 6667333

[btae276-B13] Katoh K , Rozewicki J, Yamada KD. MAFFT online service: multiple sequence alignment, interactive sequence choice and visualization. Brief Bioinform 2019;20:1160–6. 28968734 10.1093/bib/bbx108 PMC6781576

[btae276-B14] Koike-Takeshita A , Arakawa T, Taguchi H et al Crystal structure of a symmetric football-shaped GroEL: GroES2-ATP14 complex determined at 3.8 å reveals rearrangement between two GroEL rings. J Mol Biol 2014;426:3634–41. 25174333 10.1016/j.jmb.2014.08.017

[btae276-B15] Kurtzer GM , Sochat V, Bauer MW. Singularity: scientific containers for mobility of compute. PLoS One 2017;12:e0177459. 28494014 10.1371/journal.pone.0177459 PMC5426675

[btae276-B16] Li W , Godzik A. CD-HIT: a fast program for clustering and comparing large sets of protein or nucleotide sequences. Bioinformatics 2006;22:1658–9. 16731699 10.1093/bioinformatics/btl158

[btae276-B17] Lin Z , Akin H, Rao R et al Evolutionary-scale prediction of atomic-level protein structure with a language model. Science 2023;379:1123–30. 36927031 10.1126/science.ade2574

[btae276-B18] Lladós J , Cores F, Guirado F et al Accurate consistency-based MSA reducing the memory footprint. Comput Methods Programs Biomed 2021;208:106237. 34198017 10.1016/j.cmpb.2021.106237

[btae276-B19] Maes D , Zeelen JP, Thanki N et al The crystal structure of triosephosphate isomerase (TIM) from Thermotoga maritima: a comparative thermostability structural analysis of ten different TIM structures. Proteins 1999;37:441–53. 10591103

[btae276-B20] Merkel D et al Docker: lightweight linux containers for consistent development and deployment. Linux J 2014;239:2.

[btae276-B21] Mirdita M , Schütze K, Moriwaki Y et al Colabfold: making protein folding accessible to all. Nat Methods 2022;19:679–82. 35637307 10.1038/s41592-022-01488-1 PMC9184281

[btae276-B22] Mizuguchi K , Deane CM, Blundell TL et al HOMSTRAD: a database of protein structure alignments for homologous families. Protein Sci 1998;7:2469–71. 9828015 10.1002/pro.5560071126 PMC2143859

[btae276-B23] O'Sullivan O , Suhre K, Abergel C et al 3DCoffee: combining protein sequences and structures within multiple sequence alignments. J Mol Biol 2004;340:385–95. 15201059 10.1016/j.jmb.2004.04.058

[btae276-B24] Paysan-Lafosse T , Blum M, Chuguransky S et al InterPro in 2022. Nucleic Acids Res 2023;51:D418–27. 36350672 10.1093/nar/gkac993 PMC9825450

[btae276-B25] Rajapaksa S , Konagurthu AS, Lesk AM. Sequence and structure alignments in post-AlphaFold era. Curr Opin Struct Biol 2023;79:102539. 36753924 10.1016/j.sbi.2023.102539

[btae276-B26] Roca-Martínez J , Dhondge H, Sattler M et al Deciphering the RRM-RNA recognition code: a computational analysis. PLoS Comput Biol 2023;19:e1010859. 36689472 10.1371/journal.pcbi.1010859 PMC9894542

[btae276-B27] Rubio-Largo A , Castelli M, Vanneschi L et al A parallel multiobjective metaheuristic for multiple sequence alignment. J Comput Biol 2018;25:1009–22. 29671616 10.1089/cmb.2018.0031

[btae276-B28] Santus L , Garriga E, Deorowicz S et al Towards the accurate alignment of over a million protein sequences: current state of the art. Curr Opin Struct Biol 2023;80:102577. 37012200 10.1016/j.sbi.2023.102577

[btae276-B29] Taylor WR. Protein structure comparison using SAP. Methods Mol Biol (Clifton, N.J.) 2000;143:19–32. 10.1385/1-59259-368-2:19 11084900

[btae276-B30] UniProt Consortium. UniProt: the universal protein knowledgebase in 2023. Nucleic Acids Res 2023;51:D523–31. 36408920 10.1093/nar/gkac1052 PMC9825514

[btae276-B31] Varadi M , Anyango S, Deshpande M et al AlphaFold protein structure database: massively expanding the structural coverage of protein-sequence space with high-accuracy models. Nucleic Acids Res 2022;50:D439–44. 34791371 10.1093/nar/gkab1061 PMC8728224

[btae276-B32] Wierenga R. The TIM‐barrel fold: a versatile framework for efficient enzymes. FEBS Lett 2001;492:193–8. 11257493 10.1016/s0014-5793(01)02236-0

[btae276-B33] Xu Z , Horwich AL, Sigler PB. The crystal structure of the asymmetric GroEL–GroES–(adp) 7 chaperonin complex. Nature 1997;388:741–50. 9285585 10.1038/41944

[btae276-B34] Zhang Y , Skolnick J. TM-align: a protein structure alignment algorithm based on the TM-score. Nucleic Acids Res 2005;33:2302–9. 15849316 10.1093/nar/gki524 PMC1084323

[btae276-B35] Zhou Q , Yang D, Wu M et al Common activation mechanism of class a GPCRs. Elife 2019;8:e50279. 31855179 10.7554/eLife.50279 PMC6954041

