# Supplementary material for: Large-scale structure-informed multiple sequence alignment of proteins with SIMSApiper
Source: Bioinformatics. 2024 Apr 22;40(5):btae276. doi: 10.1093/bioinformatics/btae276 (PMC11099654; doi:10.1093/bioinformatics/btae276)
Supplement: btae276_Supplementary_Data [file btae276_supplementary_data.pdf]

Supplementary Material

This file contains Supplementary Figures 1-6 and Supplementary Tables 1-3, as well as the technical documentation of SIMSApiper.

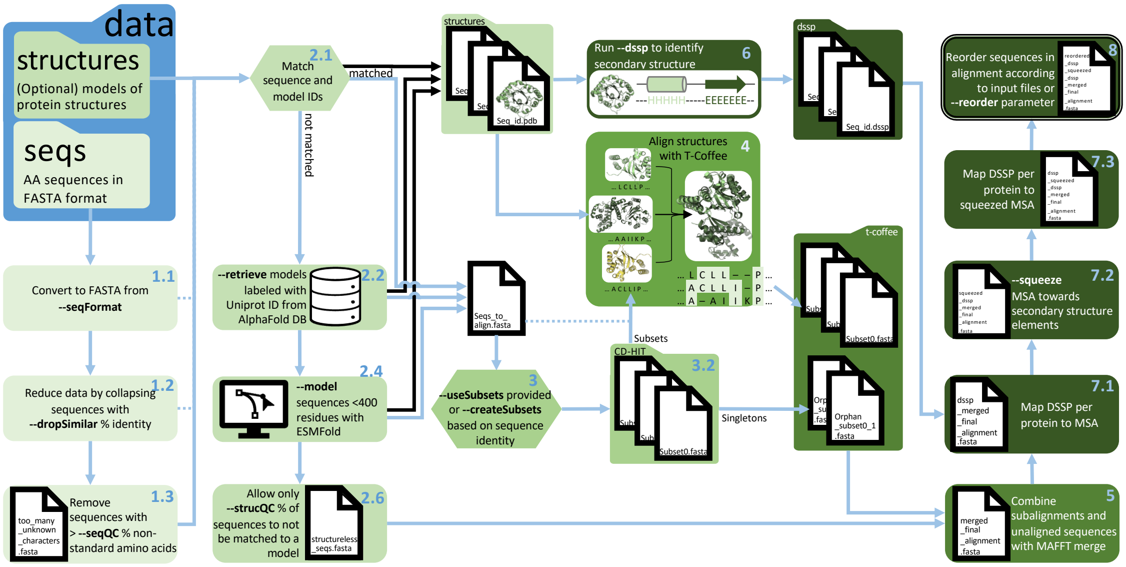

Fig. S1. Representation of data handling and tools in SIMSApiper. File and directory names match automatically generated out directory. Flags related to each step in bold. Detailed explanations for every step as well as all possible flags can be found in the Technical documentation below.

Table S1. Computer architecture used by SIMSApiper for each validation dataset. This hardware is part of the VSC Tier-2 general-purpose clusters provided by VUB-HPC. As independent steps or the alignment of the different subsets can happen in parallel, the two types of architecture available can be used depending on general cluster work load.

Architecture	HOMSTRAD	TIM-barrels	GroEL
2x 14-core INTEL E5-2680v4<br>256 GB memory<br>1 TB local storage	yes	no	yes
2x 20-core INTEL Xeon Gold<br>192 GB memory<br>1 TB local storage	yes	yes	yes

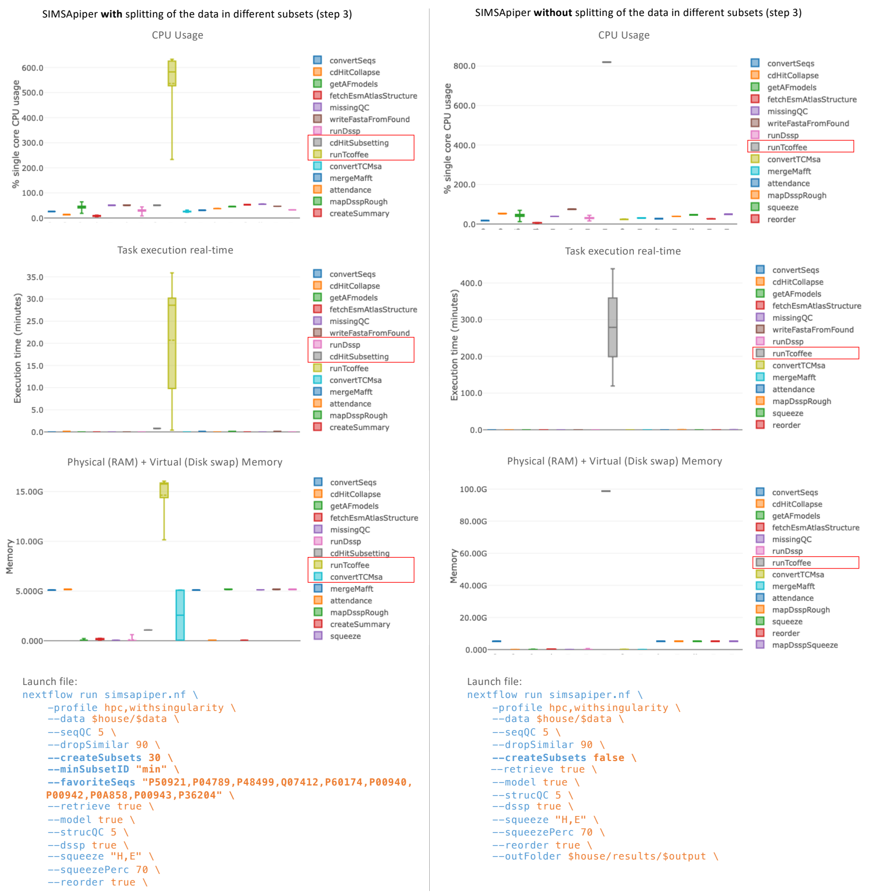

Fig. S2. Computational resources needed to generate a high-quality MSA of the TIM barrel protein family with SIMSApiper. **Left:** CPU, memory, runtime and flags needed by SIMSApiper to align 379 proteins using subsets (step 3) to speed up the alignment step (step 4). The data was split in 6 subsets based on its Sequence Identity (SI). **Right:** CPU, memory, runtime and flags needed by SIMSApiper to align 379 proteins (step 4) when step 3 is skipped. The entire dataset was submitted in one batch to T-Coffee. This results in a significant increase in required resources and highlights the importance of step 3. The plots were automatically generated by Nextflow and the values are represented by box-plots as different jobs ran in parallel to speed up the processes.

Table S2. Column Scores (CSs) between $\mathrm{MSA}_{\mathrm{SIMSApiper}}$ and $\mathrm{MSA}_{\mathrm{ref}}$ per region and overall.

Region	With squeezing	No squeezing
Conserved ($\beta\alpha$ units)	99.3%	99.3%
Unstructured (loops)	91.3%	85.2%
Overall ($\beta\alpha$ units + loops)	95.8%	93.1%

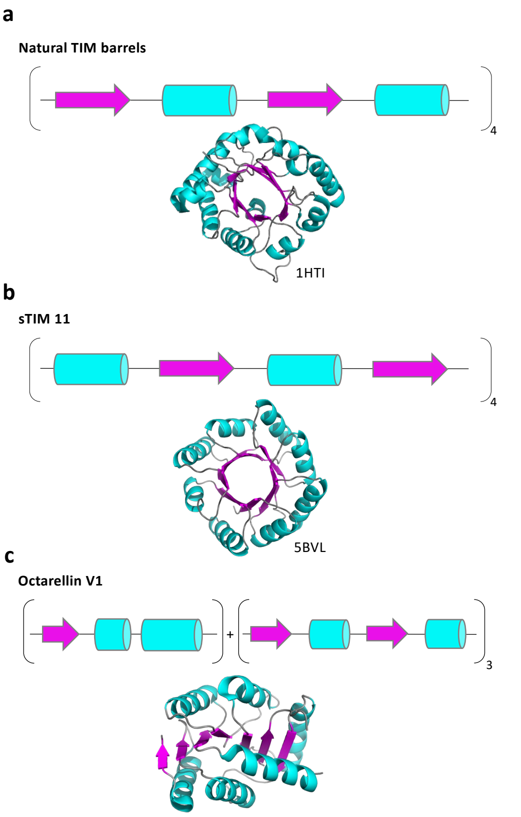

d

Natural / Designed	RMSD (Å)	Seq. identity (%)
	TPIS (1HTI)	TPIS (P60174)
sTIM 11 (5BVL)	4.4	20.0
Octarellin V1 (5BOP)	6.2	18,64

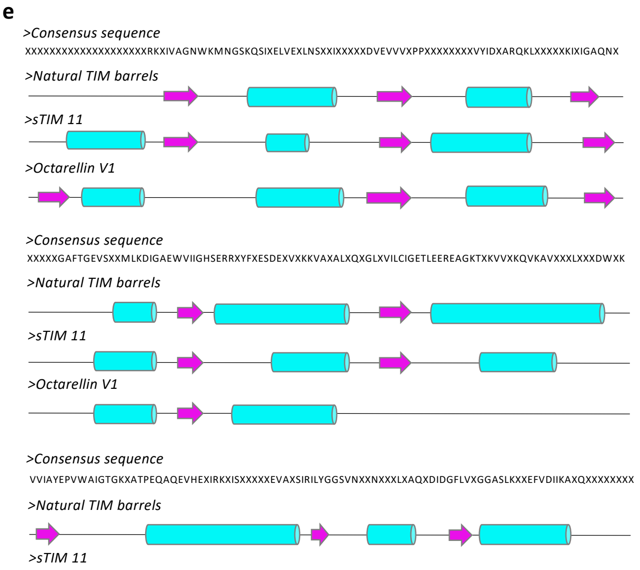

Fig. S3. Aligning highly divergent proteins with conserved secondary structure with SIMSApiper. **a) b) c)** Secondary and tertiary structure of natural and two designed TIM barrels. β-sheets are in cyan, α-helices are in magenta, loops in grey. The natural TIM barrels consist of 4 antisymmetric ($\beta\alpha\beta\alpha$) units [Wierenga, 2001], sTIM 11 consists of 4 symmetric ($\alpha\beta\alpha\beta$) [Figueroa et al., 2013] units and Octarellin V1 presents a ($\alpha\beta\alpha$) sandwich architecture [Figueroa et al., 2013]. **d)** Sequence and structural comparison of a natural TIM barrel (human TPIS, UNIPROT id:P60174, PDB id:1HTI) and two designed TIM barrels. Both at the sequence and structural level, the designed TIM barrels have relatively low identity. **e)** Alignment of the secondary structure elements of natural and designed TIM barrels obtained with SIMSApiper. SIMSApiper successfully aligned the secondary elements of the different TIM barrels

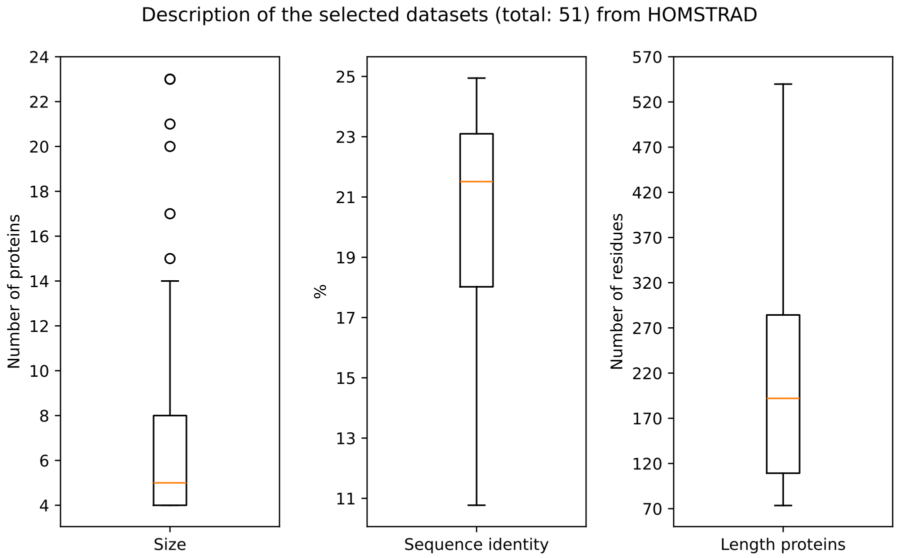

Fig. S4. The 51 most challenging datasets from HOMSTRAD (Homologous Structure Alignment Database) [Mizuguchi et al., 1998] (release date: 07/05/2022): HOM51. The datasets with more than 4 proteins and a sequence idenity (SI) <25% were selected. Every box plot shows the median, Q1, Q3, minimum, maximum and outliers.

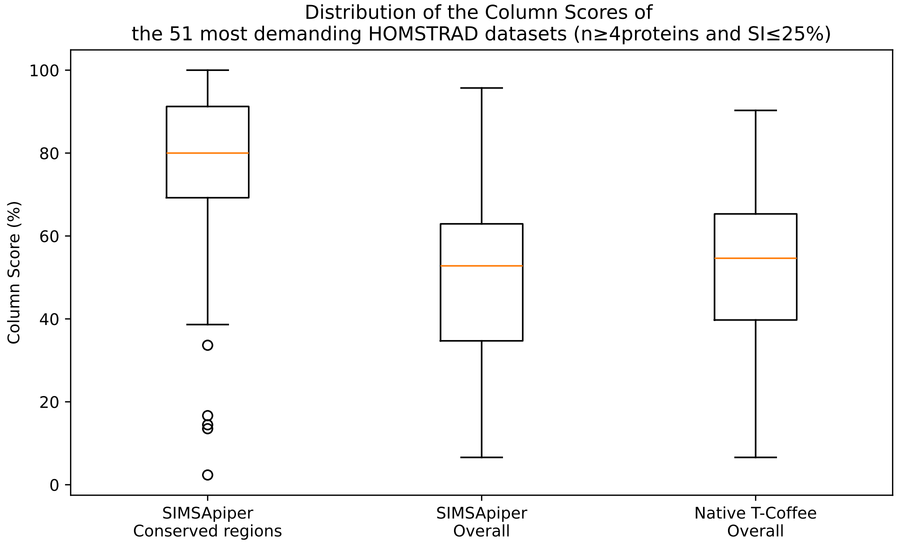

Fig. S5. Column Scores (CSs) obtained when comparing the HOM51 MSAs and the SIMSApiper MSAs. Conserved regions stands for the the regions with conserved secondary structure elements. Overall columns represents the conserved and unconserved regions together. Native T-Coffee stands for running SIMSApiper without pre- or post-processing. Every box plot shows the median, Q1, Q3, minimum, maximum and outliers.

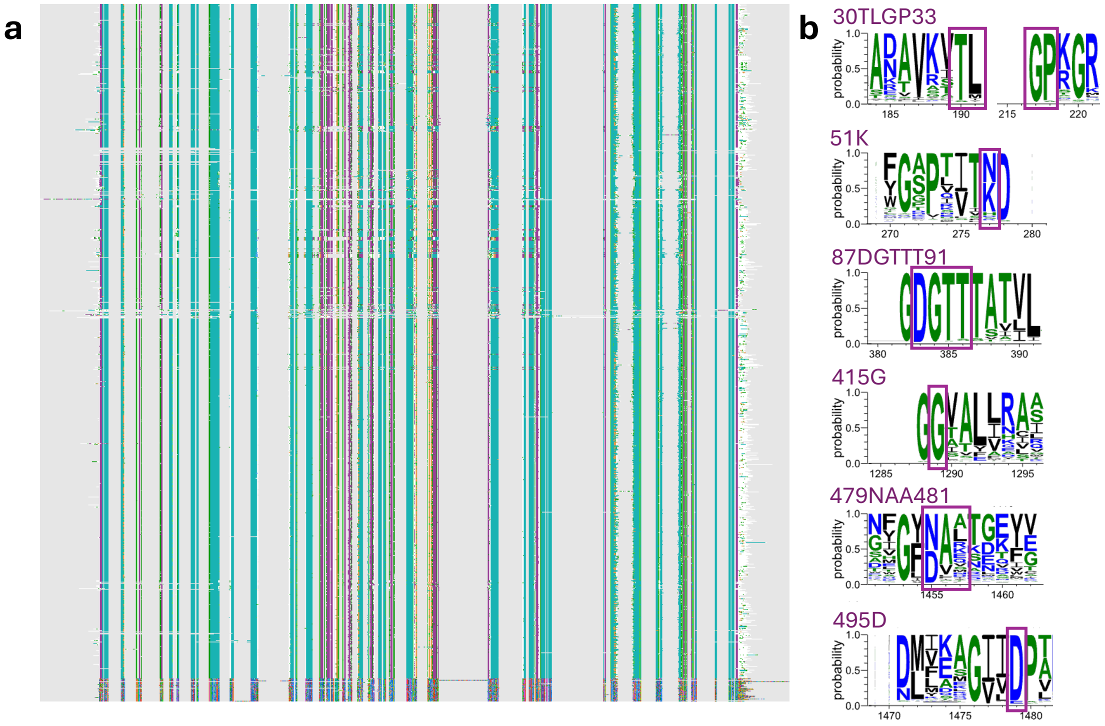

Fig. S6. Alignment of the GroEL protein family. **a)** DSSP values mapped onto the final alignment (step 7.3) show good alignment of secondary structure elements, especially for α Helix (cyan). Color scheme provided by AliView [Larsson, 2014]. **b)** Sequences logos of ATP-binding site residues (pink border) as described under Uniprot entry P0A6F5 from experimental studies [Koike-Takeshita et al., 2014, Xu et al., 1997]. Sequence logo was created with Weblogo V3.7.12 [Crooks et al., 2004].

Technical documentation

This documentation pertains to SIMSApiper version 1, published on March 20st, 2024. We continuously work on expanding and improving the pipeline. For the most current version, please visit our GitHub.

Install requirements

SIMSApiper needs a sufficient amount of scratch space and RAM (50 Sequences of 400 residues with 60% sequence identity (SI) need 30GB disk space and 10GB RAM). Prospective users need to clone the git repository using

```
git clone https://github.com/Bio2Byte/simsapiper.git
```

Further requirements include:

- Nextflow
- Java V11
- Python3

The dependencies can be retrieved 4 different ways:

- Singularity /Apptainer (-profile withsingularity)
- Docker (-profile withdocker)
- Conda (-profile withconda)
  - Select Linux or ARM-Apple .yml file in nextflow.config
  - Install T-Coffee, SAP and TMalign manually
- Local execution (-profile standard)
  - CD-Hit
  - MAFFT
  - Biopython
  - BLAST (V2.14)
  - Pandas
  - DSSP (Mac: V2.2 Linux: V3.0)
  - T-Coffee (V13.45.61.3c310a9)
  - TM-align (V20190822)
  - SAP (V1.1.3)

QuickStart

Use directory ‘toy_example‘ to test installation. SIMSAPiper will automatically recognise directories called ‘data‘ if none is specified. The directory contains:

- Subdirectory ‘seqs‘ with fasta-formatted protein sequences.
- Optional: subdirectory ‘structures‘ with 3D protein structure models.

Recommended settings can be enabled using the flag **--magic**. The pipeline can be launched using this command line or the launch file called ”align_magic.sh” to run the toy_example dataset.

```
nextflow run simsapiper.nf -profile server,withsingularity --data $(pwd)/toy_example/data --magic
```

Options and Parameters

By default most flags are set to False. Adding a flag to the command line will set it to True and activate it. Some flags can carry additional information, such as percentages or filenames. The complete list can be found table S3.

The **--magic** flag is equivalent to

```
nextflow run simsapiper.nf
    -profile server,withsingularity
    --seqFormat fasta --seqQC 5 --dropSimilar 90
    --outFolder "simsa_time_of_execution" --outName "magicMsa"
    --minSubsetID "min" --createSubsets 30
    --retrieve --model --strucQC 5
    --dssp --squeeze "H,E" --squeezePerc 80
    --reorder
```

Table S3. All avilable flags in SIMSApiper with default values and explanation of function and recommended input (type). AA stands for amino acids in this table.

Flag	Function	Default	Recommendation
-resume	Retry the last run, no rerun of completed jobs -resume [hash] to retry specific run		
-profile standard	Local execution		-profile standard,withconda
-profile server	Linux server execution		
-profile hpc	HPC execution using SLURM		
-profile withdocker	Dependencies via docker container		
-profile withsingularity	Dependencies via apptainer images		
-profile withconda	Dependencies via conda (except T-Coffee)		
--condaEnvPath	Path to conda environment (if –profile withconda)	false	create with .yml file for ARM-Apple (-profile standard) Linux (-profile server) automatically
--data	Path to data directory	data	
--structures	Path to structure files directory	**--data** /structures	
--seqs	Path to sequence files directory	**--data** /seqs	
--seqFormat	Input sequence format according to biopython	fasta	
--seqQC	Ignore sequences with % non-standard AA	5	
--dropSimilar	Collapse sequences with % SI	false	90
--favoriteSeqs	Sequence labels that need to stay in alignment	false	"SeqLabel1,SeqLabel2"
--outFolder	Set directory name for output files	results/simsa_time _of_execution	
--outName	Set final MSA file name	finalmsa	
--createSubsets	Creates subsets of maximally % SI	false	30
--minSubsetID	Minimal % SI to be in a subset	20	"min" to collate small CD-Hit clusters
--maxSubsetSize	Sets maximal number of sequences in a subset	true	$\leqslant$ 400 AA: --maxSubsetSize 100, $\geqslant$ 400 AA: --maxSubsetSize 50
--useSubsets	User provides multiple sequence files corresponding to subsets	false	Provide sequences not fitting any subset in "orphan_*.fasta"
--retrieve	Retrieve protein structure models from AF2 DB	false	
--model	Predict protein structure models with ESM Atlas	false	
--strucQC	Maximal % of sequences not matched to a 3D structure	5	
--tcoffeeParams	Additional parameters for Tcoffee	false	"--help"
--mafftParams	Additional parameters for MAFFT	false	"--localpair --maxiterate 100"
--dssp	Map DSSP code to alignment	false	
--squeeze	Squeeze alignment towards conserved 2nd structure categories	false	"H,E"
--squeezePerc	Minimal occurence % of anchor element in MSA	80	
--reorder	Order final MSA by input file order	false	
--convertMSA	Covert final MSA file from fasta to selected format	false	"clustal"
--magic	Launch a run with recommended settings	false	

Input files

Config file: Nextflow.config

The config file holds all parameters needed to adapt SIMSApiper to your system and their default values. It includes execution profiles for local, server and HPC-SLURM as well as Conda, Docker and Singularity. Standard parameters and execution profiles can be easily adapted to the users system.

Sequence Input (--seqs)

Sequence data can be presented either as one file with all sequences or multiple sequence files, which can later be used for subsetting (**--use_subsets**). Sequence data can be provided in any file format supported by the biopython package. Should the user plan to retrieve structural information via AlphaFold Database (**--retrieve**), sequence IDs should start with the Uniprot ID.

Structural input (--structures)

Structural input is optional, but if provided, it must be in .pdb format of any origin (experimentally generated, from Protein Data Bank or modelled). Sequence labels and the structure filenames must match exactly. If experimental structures are used, only one chain should be provided to SIMSApiper. Mutations or less then 5% sequence divergence between input-sequence and model-sequence will not impact the MSA quality if the mutations do not impact the overall organisation of the protein. Modelled structures by ESMF, or AlphaFold2 using ColabFold can be used to omit this constraint.

1. Preprocessing

1.1 Convert Sequence Files:

Parse sequence files using biopython to provide sequences in fasta-2-line format.

1.2 Reduce dataset size (--dropSimilar):

Calculation time scales exponentially with number of input sequences. We therefore recommend at least a cutoff of 90% SI to remove redundant sequences or 70% to have a more general overview of the protein family and address sampling bias. SIMSApiper uses CD-Hit to cluster the input sequences and keeps cluster representatives in the dataset to align. Proteins inside clusters will be excluded from the downstream processes, but important proteins (such as reference sequences) can be added to the representatives with **--favoriteSeqs**.

1.3 Quality control for input sequences (--seqQC):

Too many non-standard amino acids can result in a poor alignment, we therefore remove all sequences containing more than **--seqQC** % non-standard or unresolved amino acids from the process.

2. Match sequence with structure information

2.1 Identify missing models:

SIMSApiper matches sequence ID and structure model filenames, and compiles a list of all protein sequences that have no structural information available.

2.2 Retrieve models from AlphaFold2 Protein Structure Database (--retrieve):

AlphaFold2 models are considered the most accurate protein structure predictions, and for most Uniprot entries there already exists a pre-computed model in the AF2 DB. Where possible, we enable users with SIMSApiper to avoid re-computing existing models and collect these models automatically for them.

2.3 Identify missing models:

The list gets updated and all proteins for which SIMSApiper could find model in AF2 DB are removed.

2.4 Model Sequences with ESMF (--model):

The ESM Atlas provides very fast prediction of novel protein structure models from sequence without the need for Uniprot ID-labelled sequences. We use the ESM Atlas API to submit all sequences shorter then 400 residues and collect the resulting models.

2.5 Identify missing models:

The list gets updated and all proteins for which SIMSApiper could generate a model with ESMF are removed.

2.6 Assess number of structure models and sequences to be aligned (--strucQC):

After these rounds of data collection, the final amount of structural information is assessed. The final alignment quality decreases if too many sequences are not matched to a model, and we therefore establish a minimal cutoff. SIMSApiper fails if there is too many missing models before the time-intensive T-Coffee step to allow users to add more information manually or continue the run with a more permissive cutoff. Sequences not matched to a model are collected in a separate file to aid this.

3. Subsetting - Division of the dataset into different subsets

Calculation time and space requirements of T-Coffee alignments scale exponentially with the number of sequences and the length of these sequences. We therefore established two presets: For an average sequence length shorter than 400 residues, maximally 100 sequences per subset are permitted. For longer sequences we suggest a cutoff of 50. Users can also set a custom maximum number of members per cluster with **--maxSubsetSize**. Sequences that do not fit with any subset can be labelled orphan, and will later be aligned to the T-coffee alignments with MAFFT based on sequence information.

3.1 User-provided subsets (--useSubsets):

Users can generate these subsets based on prior knowledge on function, phylogenetic relationships or SI and provide these subsets in as separate sequence files to SIMSApiper. These file will still be filtered to only include sequences for which a model has been found.

3.2 Automatically generated subsets (--createSubsets):

SISMApiper can create subsets automatically with PSI-CD-HIT and a low SI cutoff. If the CD-Hit generated clusters are are too large to become subsets, we split these clusters to obtain evenly distributed clusters smaller then **--maxSubsetSize**. If there are too many sequences that do not fit in any cluster, we interactively decrease the CD-Hit SI threshold by 5% until **--minSubsetIdentity**. We observed that we obtained the best alignments when we submitted the minimal number of subsets, and provide the setting **--minSubsetIdentity "min"** to reduce overall number of subsets by collating small clusters and singletons until **--maxSubsetSize** reached.

4. Run T-Coffee (--tcoffeeParams)

T-Coffee mode 3Dcoffee is a state-of-the-art structure-informed alignment method [O'Sullivan et al., 2004, Carpentier and Chomilier, 2019]. It uses a combination of algorithms TMalign and SAP have to provide the best results [Baltzis et al., 2022]. SIMSApiper can align each subset individually, but also in parallel depending on available resources. T-Coffee parameters in this pipeline are:

```
t_coffee -in=subset_0.fasta  ${task.cpus} -ulimit=${task.memory} -outfile=aligned_subset_0.aln
    -method TMalign_pair -evaluate_mode=t_coffee_slow  -mode=3dcoffee  -pdb_min_cov=1
```

Users can append any other T-Coffee flag with **--tcoffeeParams "-quiet"**.

5. Run MAFFT (--mafftParams)

After all T-Coffee subalignments are ready, these and any orphan or structureless sequences are combined into the final alignment using MAFFT. MAFFT mode 'merge' is conserving the subalignment structure. Users can add other mafft flag or alignment modes with **--mafftParams "--localpair --maxiterate 100"**

6. Run DSSP (--dssp)

All structure models are translated into 2D secondary structure nomenclature using the DSSP codes

7. Improve MSA

7.1 Map DSSP to MSA

SIMSApiper maps DSSP sequences for each model on sequences of the MSA, conserving the gaps. We permit model sequence and alignment sequence to diverge in up to 5% of residues if the protein length is maintained (point mutations). If the length is not maintained, insertions/deletions can only appear at the C-/N-terminus. If these conditions are not met, the sequence will be excluded from this step and remains unconverted in the mapped alignment.

7.2 Squeeze MSA towards conserved secondary structure elements (--squeeze):

MSA in unstructured or less conserved regions (e.g. loops) are usually very disperse. The MAFFT-merge step additionally is prone to add many gaps to the alignment. SIMSApiper identifies conserved secondary structure categories such as helices and β-sheets with **--squeeze "H,E"**, and squeezes the MSA towards these regions. DSSP codes representing helices, i.e, H, G and I, are considered the same by SIMSApiper We also implemented a minimum of 3 consecutive conserved columns to be considered a region. The percentage threshold for region to be 'conserved' can be set with **--squeezePerc**.

7.3 Map DSSP to squeezed MSA

The alignment of secondary structure elements has been a useful tool for us to assess the quality of a MSA without a reference alignemnt. SIMSApiper maps the DSSP codes on the squeezed MSA as well to facilitate this analysis. The same constraints apply as before.

8. Reorder MSA (--reorder)

Order MSA according to the order of sequences in the input files. If more then one sequence input file is provided, the MSA will be reordered based on the order given in the files organized alphabetically. Instead of alphabetically, the user can also select explicitly the order of the files with **--reorder "gamma.fasta,delta.fasta"**.

9. Convert MSA

SIMSApiper outputs the final MSA in fasta-2-line format, but can convert to any format supported by python package biopython.

Log files

SIMSApiper provides different log files containing information about the run and resource usage, or the data analyzed.

Data report

The simsapiper_summary.md file contains information about the results of individual SIMSApiper steps, e.g. on number of sequences in the input files, excluded sequences, sequences matched and not matched to a protein structure model as well as total number of sequences in the final aligned file. It also includes explicit paths for all relevant output files.

Resource log

The file nextflow_report_outName.html and resources_outName.txt contain information on execution times and resource usage per step.

Execution log

The file outName_time.nflog is created only when using the launch file. It reports all **--flag** settings and captures terminal output of Nextflow pipeline for error tracing, as well as the unique execution hash and flags for resuming the specific job.

References

A. Baltzis, L. Mansouri, S. Jin, B. E. Langer, I. Erb, and C. Notredame. Highly significant improvement of protein sequence alignments with AlphaFold2. *Bioinformatics*, 38(22):5007–5011, Nov. 2022.

M. Carpentier and J. Chomilier. Protein multiple alignments: Sequence-based versus structure-based programs. *Bioinformatics*, 35(20):3970–3980, Oct. 2019.

G. E. Crooks, G. Hon, J.-M. Chandonia, and S. E. Brenner. WebLogo: A sequence logo generator. *Genome research*, 14(6): 1188–1190, 2004.

M. Figueroa, N. Oliveira, A. Lejeune, K. W. Kaufmann, B. M. Dorr, A. Matagne, J. A. Martial, J. Meiler, and C. Van De Weerdt. Octarellin VI: Using Rosetta to Design a Putative Artificial (β/α)8 Protein. *PLoS ONE*, 8(8):e71858, Aug. 2013.

A. Koike-Takeshita, T. Arakawa, H. Taguchi, and T. Shimamura. Crystal structure of a symmetric football-shaped GroEL: GroES2-ATP14 complex determined at 3.8 å reveals rearrangement between two GroEL rings. *Journal of Molecular Biology*, 426(21): 3634–3641, 2014.

A. Larsson. Aliview: A fast and lightweight alignment viewer and editor for large datasets. *Bioinformatics*, 30(22):3276–3278, 2014.

K. Mizuguchi, C. M. Deane, T. L. Blundell, and J. P. Overington. HOMSTRAD: A database of protein structure alignments for homologous families. *Protein science*, 7(11):2469–2471, 1998.

O. O'Sullivan, K. Suhre, C. Abergel, D. G. Higgins, and C. Notredame. 3DCoffee: Combining Protein Sequences and Structures within Multiple Sequence Alignments. *Journal of Molecular Biology*, 340(2):385–395, July 2004.

R. Wierenga. The TIM-barrel fold: A versatile framework for efficient enzymes. *FEBS Letters*, 492(3):193–198, Mar. 2001.

Z. Xu, A. L. Horwich, and P. B. Sigler. The crystal structure of the asymmetric GroEL–GroES–(adp) 7 chaperonin complex. *Nature*, 388(6644):741–750, 1997.
